# Antifungal Activity of Oleuropein against *Candida albicans*—The In Vitro Study

**DOI:** 10.3390/molecules21121631

**Published:** 2016-11-28

**Authors:** Nataša Zorić, Nevenka Kopjar, Ivan Bobnjarić, Igor Horvat, Siniša Tomić, Ivan Kosalec

**Affiliations:** 1 Agency for Medicinal Products and Medical Devices of Croatia (HALMED), Ksaverska cesta 4, HR-10000 Zagreb, Croatia; sinisa.tomic@halmed.hr; 2 Institute for Medical Research and Occupational Health, Ksaverska cesta 2, HR-10000 Zagreb, Croatia; nkopjar@imi.hr; 3 Department of Microbiology, Faculty of Pharmacy and Biochemistry, University of Zagreb, Schrottova 39, HR-10000 Zagreb, Croatia; ivan.bobnjaric@gmail.com (I.B.); igor.horvat@gmail.com (I.H.); ikosalec@pharma.hr (I.K.)

**Keywords:** oleuropein, antifungal activity, *Candida albicans*, virulence factors

## Abstract

In the present study we investigated activity of oleuropein, a complex phenol present in large quantities in olive tree products, against opportunistic fungal pathogen *Candida albicans*. Oleuropein was found to have in vitro antifungal activity with a minimal inhibitory concentration (MIC) value of 12.5 mg·mL^−1^. Morphological changes in the nuclei after staining with fluorescent DNA-binding dyes revealed that apoptosis was a primary mode of cell death in the analyzed samples treated with subinhibitory concentrations of oleuropein. Our results suggest that this antifungal agent targets virulence factors essential for establishment of the fungal infection. We noticed that oleuropein modulates morphogenetic conversion and inhibits filamentation of *C. albicans*. The hydrophobicity assay showed that oleuropein in sub-MIC values has significantly decreased, in both aerobic and anaerobic conditions, the cellular surface hydrophobicity (CSH) of *C. albicans*, a factor associated with adhesion to epithelial cells. It was also demonstrated that the tested compound inhibits the activity of SAPs, cellular enzymes secreted by *C. albicans*, which are reported to be related to the pathogenicity of the fungi. Additionally, we detected that oleuropein causes a reduction in total sterol content in the membrane of *C. albicans* cells, which might be involved in the mechanism of its antifungal activity.

## 1. Introduction

*Candida* species are commensal organisms that normally colonize mucosal surfaces of healthy individuals and, under conditions of host weakness, can become opportunistic pathogens. Among *Candida* species, *Candida albicans* is the predominant cause of invasive fungal infections; however, in recent years, a growing incidence of infections caused by non-*albicans* species has been observed [1]. An increase in serious human infections in immunocomprised patients caused by fungi and a progression of drug resistance to conventional therapeutics triggered a need for more effective treatment.

Several studies have reported that olive leaf extract and its constitutes, particularly oleuropein and hydroxytyrosol, have health benefits, including antioxidant and antimicrobial properties [2,3,4,5].

Oleuropein was found to inhibit the growth of *Staphylococcus aureus*, *Bacillus subtilis*, and *Psudomonas solanecearum* [6]. It was shown that it also inhibits germination and sporulation of *Bacillus megaterium* [7], and an outgrowth of germinating spores of *Bacillus cereus* [8]. The activity of oleuropein was investigated in vitro against *Mycoplasma hominis*, *M. fermentas*, *M. pneumoniae*, and *M. pirum*. Oleuropein inhibited mycoplasmas at concentrations from 20 to 320 mg·L^−1^ [9]. Considering that molecules that derive from natural sources have considerable antifungal properties and can be a promising source for the development of new anti-candidal therapy [10], we performed in vitro tests to examine the effect of this phenolic compound on *C. albicans*, one of the most important opportunistic fungal pathogen, and to investigate its possible mechanism of action.

## 2. Results and Discussion

Antifungal susceptibility testing was used to estimate the drop of viability up to 90% in comparison to the control (untreated cells). Our findings showed that minimal inhibitory concentration (MIC) of oleuropein against *C. albicans* was 12.5 mg·mL^−1^.

A quantitative fluorescent-dye exclusion assay revealed (Table 1) that oleuropein significantly (*p* < 0.05, Pearson chi-square test) reduced cell viability compared to the negative control at certain applied concentrations (12.5 mg·mL^−1^; 1.25 mg·mL^−1^; 0.195 mg·mL^−1^).

**Figure 1:**
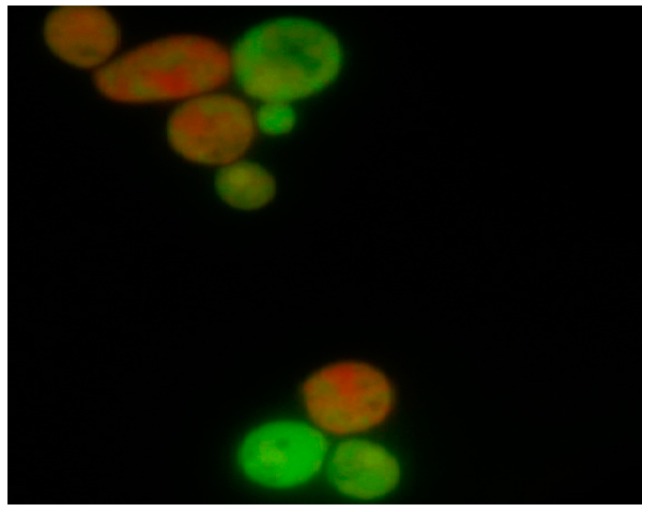
Appearance of *C. albicans* blastospores treated with oleuropein following staining with ethidium bromide and acridine orange according to the fluorescent-dye exclusion method: viable normal blastospores excluded ethidium bromide, and their nuclei were bright green with an intact structure. Non-viable cells had orange to red colored chromatin with organized structure. Apoptotic cells were bright green with highly condensed or fragmented nuclei.

Analysis of *C. albicans* cells following 18 h in vitro exposure to oleuropein indicated a cytotoxic effect of oleuropein that was concentration-dependent. Further, intergroup comparison of viable vs. dead cells using the Pearson chi-square test revealed statistically significant differences (*p* < 0.05) between tested concentrations. In the samples treated with concentrations, 1.25 mg·mL^−1^ and 0.195 mg·mL^−1^ of oleuropein apoptosis was a predominant type of cell death. Differentiation between viable and dead cells after treatment with the test agent and the altered morphology of the nuclear chromatin visualized by fluorescence microscopy is shown in Figure 1.

In order to understand mode of action of antifungal agent, it is necessary to investigate its effect on virulence factors, which are essential for development of infection in the host. It was believed in the past that yeasts passively participate in the process of pathogenesis and the establishment of fungal infection. An immunocomprised host was considered the only mechanism responsible for the establishment of opportunistic infection. Today, it is known that yeasts actively participate in the pathophysiology of the disease using mechanisms called virulence factors [10]. The main advantages of targeting virulence are a higher number of potential targets for novel antifungal therapeutics, the preservation of host microbioma, and weaker selective pressure for the development of antibiotic resistance [11].

*C. albicans* is a polymorphic fungus and is able to undergo reversible morphological transition between yeast and filamentous forms. It has been reported that the growth of hyphae promotes virulence and plays an important function in tissue invasion and resistance to phagocytosis [12]. This morphological change occurs in response to external stimuli, including nutrient availability, high temperature, pH, and the presence of host macrophages [13].

We evaluated the inhibitory effect of oleuropein under hyphal-inducing conditions. For the test, we used complex media containing 10% serum, which is the most potent inducer of the hyphal morphological state [13].

**Figure 2:**
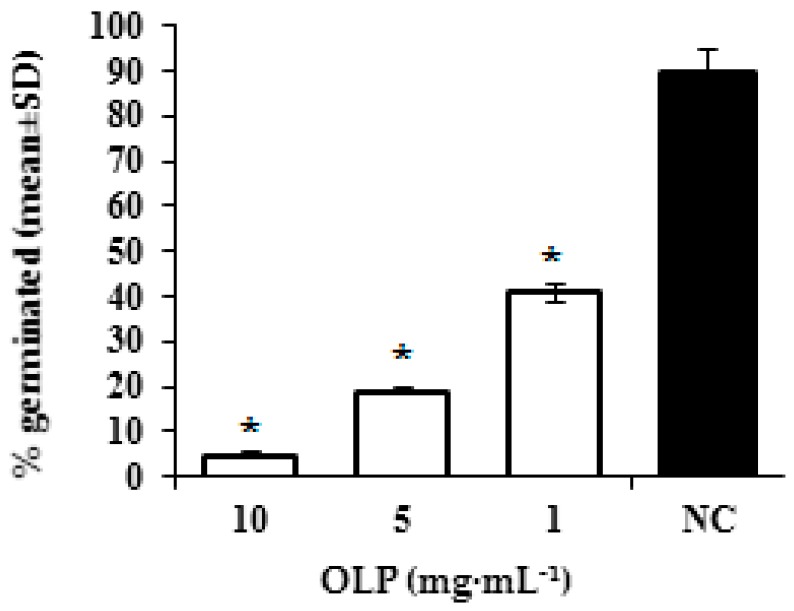
Effect of different concentrations of oleuropein (OLP) on germ-tube formation in *C. albicans*; NC—intact cells. The data are shown as means ± SD from three independent experiments (* *p* < 0.05 in comparison to NC).

After incubation at 35 °C for 3 h, statistically significant (*p* < 0.05) inhibition of morphological transition of *C. albicans* cells to filamentous form was observed for samples treated with subinhibitory concentrations (10 mg·mL^−1^, 5 mg·mL^−1^, and 1 mg·mL^−1^) of oleuropein in comparison to the negative control. The results presented in Figure 2 show the modulation of the morphogenetic conversion of *C. albicans* under the influence of oleuropein.

Important virulence factors that facilitate pathogenicity of *Candida* are host recognition, which enables the pathogen to bind to the host cells and proteins, and adherence to host surfaces. Additionally, *Candida* species can adhere to the surfaces of medical devices and form biofilms. The initial attachment of *Candida* cells is mediated by non-specific factors (hydrophobicity and electrostatic forces) and promoted by specific adhesins on the surface of fungal cells [14].

Studies have suggested that cell surface hydrophobicity is involved in adherence to epithelial cells and is associated with pathogenic potential of the yeasts [15].

**Figure 3:**
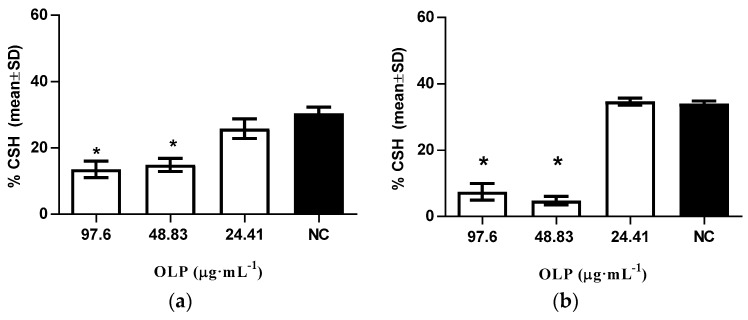
Effect of different concentrations of oleuropein (OLP) on modulation of cellular surface hydrophobicity in (**a**) aerobic conditions; and (**b**) anaerobic conditions; NC-intact cells. The data are shown as means ± SD from three independent experiments (* *p* < 0.05 in comparison to NC).

The hidrophobicity assay in our study showed that oleuropein below the MIC value induced a statistically significant change in CSH levels (*p* < 0.05), in comparison to the control for *C. albicans*, after 24 h of incubation aerobically at 25 °C by decreasing hydrophobicity from 34.05% ± 1.85% to 13.55% ± 2.46% at a concentration of 97.6 μg·mL^−1^ and from 34.05% ± 1.85% to 14.92% ± 1.99% at a concentration of 48.83 μg·mL^−1^ of oleuropein (Figure 3a). It was noted that oleuropein has a stronger influence on the decrease of CSH levels when samples are incubated 24 h at 25 °C under 10% CO_2_ conditions (Figure 3b). A statistically significant decrease in hydrophobicity under anaerobic conditions in comparison to the control was observed at an oleuropein concentration of 97.6 μg·mL^−1^ (from 34.05% ± 1.85% to 7.44% ± 2.54%) and a concentration of 48.83 μg·mL^−1^ (from 34.05% ± 1.85% to 4.78% ± 1.33%).

Most therapies for fungal infections target the ergosterol biosynthesis pathway or its end product ergosterol. This membrane sterole is unique to fungi, and is necessary for growth and the normal membrane function of fungal cells. The primary mechanism of action by which, for example, commonly used azole antifungal drugs inhibit yeast cell growth is through disruption of the normal sterol biosynthetic pathway, leading to a reduction in ergosterol biosynthesis [16].

**Figure 4:**
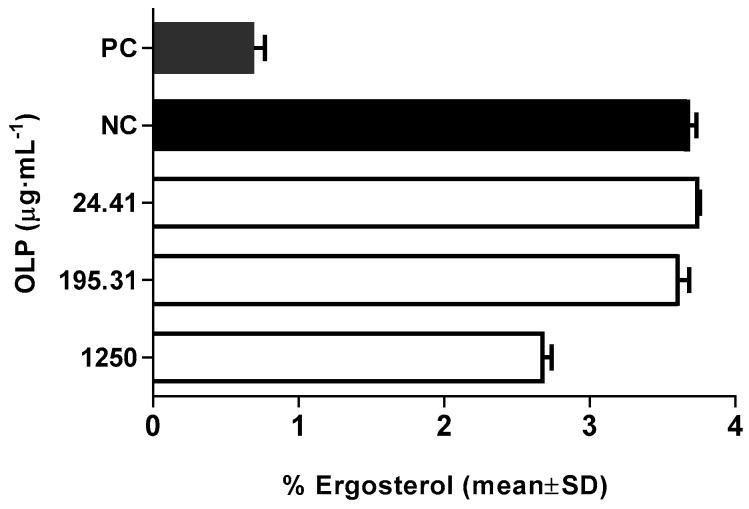
Modulation of ergosterol content at different concentration of oleuropein (OLP); PC-amphotericin 1 μg·mL^−1^; NC-intact cells. Results represent the mean of three experiments ± SD (*p* < 0.05 in comparison to NC).

We tested the effect of oleuropein on the membrane of *C. albicans* cells using an ergosterol synthesis assay. Figure 4 shows the modulation of ergosterol biosynthesis at different concentrations of oleuropein.

Analysis showed that, at subinhibitory concentrations, our phenolic component had altered sterol content and subsequently affected the cell membrane. Further, our date indicated that the compound decreased ergosterol content in a dose-dependent fashion. Intergroup comparisons revealed statistically significant differences (*p* < 0.05) between tested concentrations. At the highest concentration (1.25 mg·mL^−1^), oleuropein caused a 28% reduction in total sterol content.

**Figure 5:**
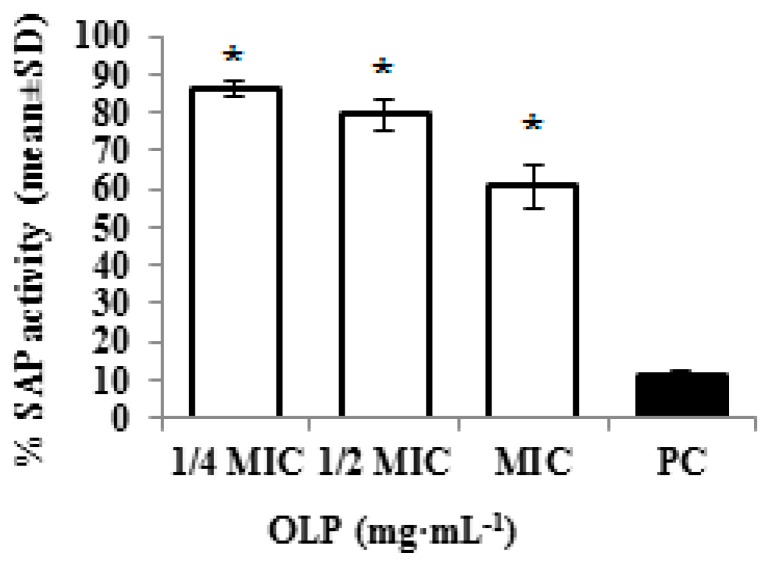
SAP activity of *C. albicans* treated with different concentrations of oleuropein OLP (1/4 MIC—3.12 mg·mL^−1^, 1/2 MIC—6.25 mg·mL^−1^, MIC—12.5 mg·mL^−1^); PC—pepstatin 0.1·mg·mL^−1^. Results represent the mean of three experiments ± SD (* *p* < 0.05 in comparison to PC).

Additional *Candida* factors important in adherence, tissue penetration, invasion, and destruction of host tissue are extracellular hydrolytic enzymes [17]. The most significant hydrolytic enzymes involved in the virulence of *C. albicans* are SAP proteins [18]. Their relationship with the virulence has been demonstrated by the degradation of cellular substrates such as proteins related to immunological and structural defenses of the host [19]. The present study revealed that the cultivation of *C. albicans* in the presence of oleuropein caused a statistically significant (*p* < 0.05) inhibition of SAP activity (Figure 5).

It should be noted that the effect of oleuropein on the inhibition of SAP activity was dose-dependent. Oleuropein caused approximately 13% of inhibition at a concentration of 3.12 mg·mL^−1^ (1/4 MIC), 20% at a concentration of 6.25 mg·mL^−1^ (1/2 MIC), and 30% at a concentration of 12.5 mg·mL^−1^ (MIC). Results from the present study suggest that oleuropein inhibition of SAP activity might be related to decreased pathogenicity of *C. albicans*.

In conclusion, the findings of our study show that oleuropein has promising in vitro activity against *C. albicans*. This antifungal agent targets virulence factors essential for the establishment of opportunistic infection. Additional studies are necessary to further investigate the mechanisms of action of oleuropein and the possible development of a new antifungal therapeutic.

## 3. Materials and Methods

### 3.1. Materials

*Candida albicans* strains from the stock culture collection of the Department of Microbiology, Faculty of Pharmacy and Biochemistry, University of Zagreb, were used for all tests performed in this study. Oleuropein (Extrasynthese, Genay, France) was dissolved in a pH 7.4 phosphate buffer to prepare stock solution at a concentration of 50 mg·mL^−1^. All other chemicals and reagents, unless otherwise specified, were purchased from Sigma (St. Louis, MO, USA).

### 3.2. Methods

#### 3.2.1. Antimicrobial Susceptibility Testing

The minimum inhibitory concentration (expressed as MIC 90%) of oleuropein against *C. albicans* ATCC 10231 was assessed using the EUCAST Def. 7.3 procedure [20]. Serial broth microdilution of oleuropein in RPMI 1640% + 2% glucose (*w*/*v*) from 25 mg·mL^−1^ to 12.21 μg·mL^−1^ was performed in a sterile flat-bottom 96 well microtiter plate inoculated with 100 μL of *C. albicans* suspension adjusted to cell density of 0.5 McFarland units (Nephelometer, bioMerioux, France). The plate was incubated aerobically for 24 h at 35 °C. After incubation, 10 μL samples from each dilution were transferred to Sabouraud 2% (*w*/*v*) glucose agar and further incubated for 48 h at 35 °C. Control wells contained 100 μL of cell suspension and oleuropein solvent.

#### 3.2.2. Identification of Apoptotic and Necrotic Cells Due to Loss of Membrane Integrity

Differentiation between viable and dead cells of *C. albicans* ATCC 10231 was determined using the fluorescent dye exclusion method [21]. One hundred microliters of inoculum suspension (1.5 McFarland units) were mixed with 900 μL of RPMI 1640 (with 2% of glucose) containing oleuropein in concentrations of 12.5 mg·mL^−1^, 1.25 mg·mL^−1^, and 0.195 mg·mL^−1^. Amphotericin (1 μg·mL^−1^) served as a positive control. The samples were incubated at 35 °C for 3 h. DNA-binding dyes (ethidium bromide and acridine orange) were added to the samples at a final concentration of 100 μg/mL (1:1; *v*/*v*), and samples were analyzed. Three classes of cells were observed: viable, apoptotic, and necrotic cells.

#### 3.2.3. Inhibition of Germ-Tube Formation

The test organism *C. albicans* ATCC 10231 was cultured on Sabouraud 2% (*w*/*v*) glucose agar (Merck, Darmstadt, Germany) for 24 h at 37 °C, aerobically. Inoculum suspension (0.5 McFarland units) for the assay was prepared from fresh culture in physiological saline. The analysis was performed according to the method of Zuzarte and colleagues [22] with slight modifications. Briefly, test tubes contained 100 μL of inoculum suspension and 900 μL of 10% (*v*/*v*) fetal bovine serum (FBS) with 10 mg·mL^−1^, 5 mg·mL^−1^, and 1 mg·mL^−1^ oleuropein. The negative control contained no oleuropein. The samples were incubated at 35 °C for 3 h. Three hundred cells were counted in a Neubauer chamber using phase-contrast microscopy and the number of yeast cells with germ-tubes versus non-germinated cells were calculated.

#### 3.2.4. Modulation of Cellular Surface Hydrophobicity Levels

Modulation of cellular surface hydrophobicity (CSH) levels were assessed according to the method reported by Ishida et al. [23]. Inoculum suspension was prepared from fresh cultures of *C. albicans* ATCC 10231 in a pH 7.4 phosphate buffer with optical density of 0.5 ± 0.05 at 620 nm. Yeasts were treated with 97.6 μg·mL^−1^, 48.83 μg·mL^−1^, and 24.41 μg·mL^−1^ of oleuropein and incubated at 25 °C for 24 h. Additionally, incubation was also carried out at 25 °C for 24 h under 10% CO_2_ conditions. Following incubation, 1.2 mL of treated yeast suspension was added to 0.6 mL of xylene, mixed by vortexing for 30 s, and left for 10 min at room temperature until the 2 phases separated. The aqueous phase of the sample was measured in a spectrophotometric 96-well plate reader (iEMS Reader, Labsystem, Finland) at 620 nm and hydrophobicity was calculated using the following equation:

(A_control_ − A_test_) × 100/A_control_ (1)

where A_control_ is the optical density before treatment, and A_test_ is the optical density after treatment.

#### 3.2.5. Modulation of Membrane Ergosterol Content

The inhibition of ergosterol synthesis was determined according to the method of Arthington-Skaggs et al. [24] in inoculums prepared from fresh cultures of *C. albicans* ATCC 10231 treated with different concentrations of oleuropein (24.41 μg·mL^−1^, 195.31 μg·mL^−1^, and 1250 μg·mL^−1^). The samples were incubated at 37 °C for 18 h on an orbital shaker (170 rpm) aerobically. The sample treated with amphotericin served as a positive control (1 μg·mL^−1^). Following incubation, the cells were harvested by centrifugation (2700× *g*, 5 min), and the weight of the cell pellet was determined. Three milliliters of freshly prepared alcoholic potassium hydroxide solution (25% *m/v*) was added to each pellet and vortexed vigorously for 1 min. Obtained cell suspensions were transferred to borosilicate glass tubes and incubated for one hour at 85 °C in a water bath and then allowed to cool. The sterol extraction was enabled by the addition of water:*n*-heptane mixture (1:3 *v*/*v*) followed by vortexing for 3 min. The produced heptane layer was transferred to a new borosilicate glass tube with a screw cap and stored (−20 °C). Prior to scanning, 0.6 mL of sterol extract was diluted in 100% ethanol (1:5) and scanned between 240 and 300 nm at 5 nm (Varian Cary 1 UV-Vis spectrophotometer, Agilent, Santa Clara, CA, USA). The ergosterol content was calculated as a percentage of the wet weight of the cell using the following equations:

%ergosterol + %24(28) DHE = [(A_281.5_/290) × F]/cell mass (2)

%24(28) DHE = [(A_230_/518) × F]/cell mass (3)

%ergosterol = [%ergosterol + %24(28) DHE] − %24(28) DHE(4)

where F is the factor of sample dilution in ethanol (1:5), and 290 and 518 are the E values (in percentages per centimeter) determined for crystalline ergosterol and 24(28) DHE, respectively.

#### 3.2.6. Inhibition of Secreted Aspartyl Proteinase Activity

The influence of oleuropein on the secreted aspartyl proteinases (SAP) activity of *C. albicans* was evaluated according to the method described by Yordanov et al. [25]. Fresh culture of vaginal clinical isolate of *C. albicans* MFBF 11103 from the stock culture collection of the Department of Microbiology, Faculty of Pharmacy and Biochemistry, University of Zagreb, was used for the test. The secretion of aspartyl proteinases was induced by adding *C. albicans* suspension (0.5 McFarland units) to a BSA-Remold liquid medium (2% (*m/v*) glucose, 0.1% (*m/v*) KH_2_PO_4_, 0.5% (*m/v*) MgSO_4_, 0.7% (*m/v*) YNB, and 1% (*m/v*) BSA (without (NH_4_)_2_SO_4_ and amino acids)). The samples were cultivated at 27 °C for 7 days on orbital shaker (150 rpm) aerobically. Following cultivation, cells were removed, and filtrated culture supernatant was used to evaluate the influence of oleuropein on the enzyme activity. Test tubes contained 0.2 mol·L^−1^ sodium citrate–HCl buffer, 1% BSA in the same buffer, culture supernatant, and oleuropein in different concentrations (12.5 mg·mL^−1^, 6.25 mg·mL^−1^, and 3.12 mg·mL^−1^). The sample treated with protease inhibitor pepstatin A dissolved in 5% DMSO served as a positive control. Test tubes with mixtures were incubated at 37 °C for 60 min (T_60_), and the reaction was terminated with 20% trichloroacetic acid. An additional control was prepared by adding 20% trichloroacetic acid to all ingredients simultaneously (T_0_). Following centrifugation at 3000× *g* for 30 min, 160 μL of clear supernatant was mixed with 40 μL of dye reagent concentrate Coomassie Brilant Blue G-250, and the optical density (OD) at 595 nm was measured. Protease activity was calculated as the difference in the OD: T_60_ − T_0_.

## 4. Statistical Analysis

All experiments were performed as triplicates at three independent occasions. Presented values are the mean and standard deviation.

Statistical significance of the data was evaluated using a one-way ANOVA with Dunnett’s post test and an X^2^ test. The level of statistical significance was set at *p* < 0.05.

## Figures and Tables

**Table 1:** Results of the quantitative fluorescent assay for simultaneous identification of apoptotic and necrotic cells due to the loss of membrane integrity in *Candida albicans* ATCC 10231 treated with oleuropein in vitro for 18 h.

Sample	Viable Cells (%)		Non-Viable Cells	
		Σ	Apoptosis (%)	Necrosis (%)
OLP_1_	16.3 ± 2.1	83.7 ± 2.1 ^OLP2,OLP3,NC^	39.3 ± 11.2 ^OLP2,OLP3,NC,PC^	44.3 ± 10.2 ^OLP2,OLP3,NC,PC^
OLP_2_	61.3 ± 6.0	38.7 ± 6.0 ^OLP3,NC,PC^	29.0 ± 4.6 ^OLP3,NC,PC^	9.7 ± 1.5 ^NC^
OLP_3_	76.0 ± 6.6	24.0 ± 6.6 ^NC,PC^	14.7 ± 5.5 ^NC,PC^	9.3 ± 1.5 ^NC^
PC	11.7 ± 3.2	88.3 ± 3.2 ^NC^	78.7 ± 0.6 ^NC^	9.7 ± 3.8 ^NC^
NC	97.3 ± 0.6	2.7 ± 0.6	1.0 ± 1.0	1.7 ± 1.1

Three hundred cells per sample per each experimental point were analyzed. Mean values ± SD are shown. OLP—concentration of oleuropein (OLP_1_—12.5 mg·mL^−1^, OLP_2_—1.25 mg·mL^−1^, OLP_3_—0.195 mg·mL^−1^); PC—positive control; NC—negative control (RPMI). Statistical significance of data was evaluated using a χ^2^ test. The level of statistical significance was set at *p* < 0.05. The abbreviations next to the means indicate from which groups the relevant group differs with statistical significance.
